# Craniovertebral and Craniomandibular Changes in Patients with Temporomandibular Joint Disorders after Physiotherapy Combined with Occlusal Splint Therapy: A Prospective Case Control Study

**DOI:** 10.3390/medicina58050684

**Published:** 2022-05-21

**Authors:** Marcin Derwich, Lawrence Gottesman, Karolina Urbanska, Elzbieta Pawlowska

**Affiliations:** 1 ORTODENT, Specialist Orthodontic Private Practice in Grudziadz, 86-300 Grudziadz, Poland; 2 Independent Researcher, New York, NY 11570, USA; tmjwizardofjaws@gmail.com; 3 Independent Researcher, 87-100 Torun, Poland; rehurbanska@gmail.com; 4 Department of Orthodontics, Medical University of Lodz, 90-419 Lodz, Poland; elzbieta.pawlowska@umed.lodz.pl

**Keywords:** temporomandibular joint disorders, occlusal splints, physiotherapy, cervical vertebrae, cervical functional spaces

## Abstract

*Background and Objectives*: The aim of the study was to assess the craniovertebral and craniomandibular changes in patients diagnosed with temporomandibular joint disorders (TMD) after physiotherapy combined with occlusal splint therapy. *Materials and Methods*: There were forty patients (32 females, 80%), diagnosed with TMD, included into the study group. After the initial series of physiotherapy, patients received maxillary occlusal splints to be worn day and night. Participants continued physiotherapy simultaneously with occlusal splint therapy for 6 months. Lateral cephalograms taken in natural head position before and after the end of the therapy were used for measurements. The control group consisted of 15 healthy participants (12 females, 80%), who had taken lateral cephalograms twice, and did not receive any type of occlusal treatment nor physiotherapy in the meantime. *Results*: Occlusal splint therapy and physiotherapy combined together significantly affected: the vertical position of the mandible (significant increase, *p* < 0.0001), the sagittal position of mandible (significant decrease, *p* = 0.0065), as well as the width of the functional space between C1 and C2 (significant decrease, *p* = 0.0042). Moreover, the cervical lordosis was restored after the end of the treatment (*p* < 0.0001). *Conclusions*: Cooperation of physiotherapists with dental practitioners is necessary in the treatment of patients with TMD, including temporomandibular joint osteoarthritis.

## 1. Introduction

Temporomandibular joint disorders (TMD) encompass different musculoskeletal diseases related to the temporomandibular joints (TMJs), adjacent muscles and bones [1,2,3]. The prevalence of TMD ranges from 5 to 12% of the general population [1,3,4] and it may even reach a frequency of 31% among adults and elderly people [5]. TMD is diagnosed most often among women [3], which is explained by the impact of estrogen signaling [6]. The prevalence peak of TMD occurs between the third and fourth decade of life [3,7]. The etiology of TMD is multifactorial. Although, the role of the dental occlusion in the etiology of TMD has been discussed by many authors, it has been summarized that there is no correlation between dental occlusion and TMD [8]. There have been found only very weak associations between dental occlusion and TMD, which are insufficient to confirm the role of occlusal interferences in the development of TMD [8,9,10]. 

TMD, including TMJ osteoarthritis (TMJ OA), should be treated with an interdisciplinary approach [3,4,11]. The major goals of the TMD treatment are: elimination of joint pain, increase of the mandibular movements’ ranges, prevention of progressing joint damage, improvement of patient’s quality of life [4]. Different methods of treatment have been presented, which can be hierarchized from the least invasive procedures (conservative methods of treatment) to the most invasive surgeries, including open joint surgeries [3,4,11]. Conservative methods of treatment include physiotherapy, occlusal splint therapy, and pharmacotherapy. It is recommended to start the TMD treatment with the least invasive procedures and, in case of failure, perform the more invasive ones [4,11].

Manual therapy, which should be performed at the beginning of the TMD treatment and then continued throughout the entire treatment, provides several positive outcomes, including, among others: decreased pain, decreased muscle spasm, increased range of motion, improved circulation, relaxation, breaking adhesions, soft tissue realignment [12]. Although physiotherapy and occlusal splint therapy have been found to decrease pain in the area of TMJs and increase maximum mouth opening [13,14,15], nothing is known about the exact changes which occur within the cervicofacial skeleton, namely the position of the mandible in relation to the cranium (craniomandibular complex), the position of the cranium in relation to the cervical spine (craniovertebral complex), as well as the changes within the curvature of the cervical spine after the end of the TMD treatment, which combines physiotherapy and occlusal splint therapy.

The connection between the cranium and cervical spine is known as the craniovertebral junction (CVJ), and consists of occiput, atlas, and axis. The motion at CVJ is biomechanically advanced and encompasses the majority of three cranial movements, namely: extension, flexion, and axial rotation [16]. Therefore, the changes within the cervical vertebrae may affect the position of head, and consequently may change the three-dimensional position of the temporal bone, which is the superior part of the TMJ.

Both the movement and the position of the mandible, which is the inferior part of the TMJ, are affected by different muscles, including not only the masticatory muscles (masseter, temporal, lateral pterygoids, medial pterygoid) [17] and the suprahyoid muscles (digastric, mylohyoid, geniohyoid) [18], but also the superior pharyngeal constrictor muscle [19]. The superior pharyngeal constrictor muscle has been found to have four parts of the origin (pteropharyngeal, buccopharyngeal, mylopharyngeal, and glossopharyngeal), and only one insertion at the posterior pharyngeal wall [19]. This indicates that the mandible is connected not only with the cranium and the hyoid bone, but also with the cervical spine, which is just behind the posterior pharyngeal wall. Consequently, the changes in the position of the cranium, the cervical spine, and the mandible are interdependent. Moreover, the changes in the morphology and position of anatomic structures, which may change the three-dimensional position of the temporal bone and the mandible, may affect the function of the TMJs.

Therefore, the aim of the study was to assess the changes in the position of cranium, mandible, and cervical vertebrae in patients diagnosed with temporomandibular joint disorders (TMD) after the end of the treatment combining occlusal splint therapy with physiotherapy.

## 2. Materials and Methods

The Medical Board Ethical Committee of Regional Medical Chamber in Gdansk, Poland (protocol code: KB-17/21) approved the study. The study was conducted with the ethical principles of the World Medical Association Declaration of Helsinki. All patients received and signed informed consent.

This research is part of the larger scientific project related to the changes that can be observed in patients diagnosed with TMD and treated with occlusal splint therapy combined with physiotherapy. This manuscript is the continuation and further evaluation of our recently published study, regarding the changes in the positions of the mandibular condyles within the glenoid fossae in patients diagnosed with TMD and treated with occlusal splint therapy combined with physiotherapy [20].

### 2.1. Design

This was a prospective case control study. All patients who had been diagnosed with TMD underwent physiotherapy and occlusal splint therapy. The duration of the treatment lasted 6 months. Lateral cephalograms (X-rays) were taken twice: before and after the end of the treatment. Healthy individuals did not undergo physiotherapy, nor occlusal splint therapy. At the end of the study, radiographs were blinded by one of the researchers (EP) so that the second researcher (MD), who analyzed the X-rays, did not know which X-ray had been taken before or after the treatment and to which patient they belonged.

### 2.2. Participants

The study was performed in the specialist orthodontic private practice in Grudziadz (Poland). Diagnostic Criteria for Temporomandibular Disorders (DC/TMD) were used to select the patients with TMD [21]. According to the DC/TMD, patients were diagnosed with at least one of the following: myalgia, arthralgia, headache attributed to TMD, disc displacement with reduction, disc displacement with reduction with intermittent locking, degenerative joint disease, subluxation [1]. The participants were at least 18 years old and not older than 65 years old. The exclusion criteria were: rheumatic disease, history of oncology treatment, traumas in the area of head and neck, pregnancy, age below 18 years old and above 65 years old, history of previous orthodontic treatment, and lack of willingness to participate in the study [20].

The control group consisted of generally healthy patients, who came for the orthodontic consultation and underwent the full process of orthodontic diagnosis, including lateral X-ray analysis. Those patients did not start the orthodontic treatment immediately after the treatment plan had been explained to them, mostly because of financial reasons. Those patients returned to the same orthodontic private practice over 1 to 2 years after the initial examination. Because of the long time that had passed since the initial examination to the recall appointment, patients underwent another set of X-rays. In the meantime, the patients did not start or complete any orthodontic or prosthodontic treatment [20].

All participants agreed and signed informed consent before they were enrolled into the study.

### 2.3. Intervention

The initial examination was performed by one of the researchers (MD) and consisted of: anamnesis, extraoral examination (palpation of: TMJs, masticatory muscles, trapezius muscles, suboccipital muscles, sternocleidomastoid muscles, and floor of the mouth), and intraoral examination (analysis of occlusion and its stability, palpation of masticatory muscles, and floor of the mouth) [20]. Lateral cephalograms were taken in all participants in maximum intercuspation, in natural head position, and with respect to the ALARA principle (as low as reasonably achievable). The lateral radiographs were taken on MyRay Hyperion X9 3D (Cefla, Imola, Italy). All of the measurements were performed with the use of Ortodoncja 9.0 Software (Ortobajt, Wroclaw, Poland).

Patients were asked to describe the pain they had felt in the area of TMJs from 0 to 3 (where: 0—no pain; 1—light pain; 2—moderate pain; 3—severe pain). Maximum mouth opening was measured intraorally with a digital caliper as a distance between the incisal edges of upper and lower central incisors in maximum opening. Both pain in the area of TMJ and maximum mouth opening were assessed twice: before and after the end of the treatment [20].

After the initial examination and the diagnosis had been performed, all of the patients were referred to the physiotherapist (KU), who specialized in the physiotherapy of the area of head and neck, including TMJs. Participants attended a series of six 1 h physiotherapy appointments, once per week for the six following weeks. There were several techniques used during physiotherapy, including: cervical spine mobilization (head traction, anterior rotation of head, mobilization of cervical vertebrae), myofascial release therapy (compressive mobilization of inferior nuchal line, compressive mobilization of muscles: masseter, temporal, sternocleidomastoid, trapezius, suboccipital, and levator scapulae), TMJ mobilization (long axis distraction, lateral distraction, passive mobilization to reduce joint sounds), and mobilization of hyoid bone (mobilization of suprahyoid and infrahyoid muscles, correction of vertical position of hyoid bone). The above-listed procedures and techniques were supported by pinopressure and dry needling in the area of head, neck, and pectoral gridle. Each patient also received a set of six exercises for autotherapy to be performed six times per day and repeated six times each (Rocabado’s 6 × 6 exercises) [21]. 

Moreover, patients also received additional exercises improving head posture, exercises strengthening TMJs, and—in case of disc subluxation—exercises with a small hose placed between the incisors to reduce the disc. There occurred small differences in the performed physiotherapy techniques among patients, depending on the patients’ individual needs.

After the end of the initial series of physiotherapy, each participant visited a dental office for bite registration before occlusal splint fabrication. Patients sat in the dental chair inclined 45° from the horizontal. First, the silicone impressions were taken. Then, the face-bow was used to transfer the position of maxilla in relation to the contractual temporomandibular joints’ axis. Finally, each patient’s mandible was manipulated to achieve hinge axis movement. Bite du jour was registered with wax (Roth power bite technique). Plaster casts were mounted in articulator and individual, acrylic occlusal splints were manufactured [20].

Each patient received a one-piece, maxillary occlusal splint. The acrylic occlusal splint was hard, and flat with incisor guidance and bilateral canine guidance, and covered the full upper dental arch. Occlusal contacts, canine guidance, and anterior guidance were analyzed with the use of an 8-micron articulating film. The process of splint adjustment and schedule of appointments (both physiotherapy and with occlusal splint) were described in detail in our previous study [20].

Having completed the 6-month period of physiotherapy combined with occlusal splint therapy, all participants were examined once again. Lateral cephalograms were taken in natural head position.

### 2.4. Outcome Measures

The primary outcomes of the study were: to measure vertical (NL/ML angle) and sagittal (Wits and ANB angle) changes in the position of the mandible after occlusal splint therapy combined with physiotherapy in patients diagnosed with TMD.

The secondary outcomes were: to assess the position of the cranium (on the basis of the craniovertebral angle), changes in the position of the cervical vertebrae, and changes in the size of functional spaces between the cranial base and the first and second cervical vertebrae after occlusal splint therapy combined with physiotherapy in patients diagnosed with TMD.

Table 1 presents reference points, lines, and angles used in cephalometric analysis to assess the changes in cervicofacial skeleton after occlusal splint therapy combined with physiotherapy in patients diagnosed with TMD [22,23].

**Figure 1:**
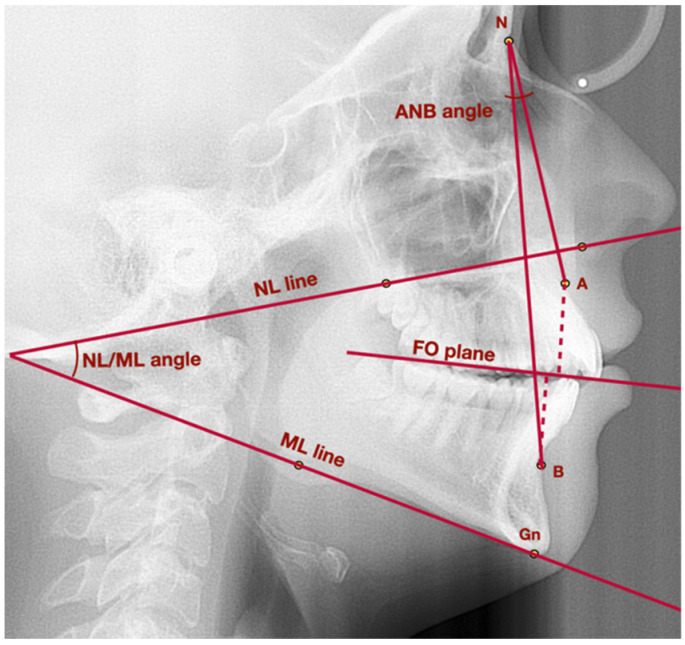
Lateral cephalogram with marked points, lines, and angles presented in Table 1 used to assess vertical and sagittal position of the mandible. A—point A; ANB—angle between lines NA and NB; B—point B; Gn—gnathion; FO plane—functional occlusal plane; ML—mandibular line; N—nasion; NL—nasal line; NL/ML—angle between lines: NL and ML.

Figure 1 presents lateral cephalogram with marked points, lines, and angles presented in Table 1 used to assess vertical and sagittal position of the mandible.

**Figure 2:**
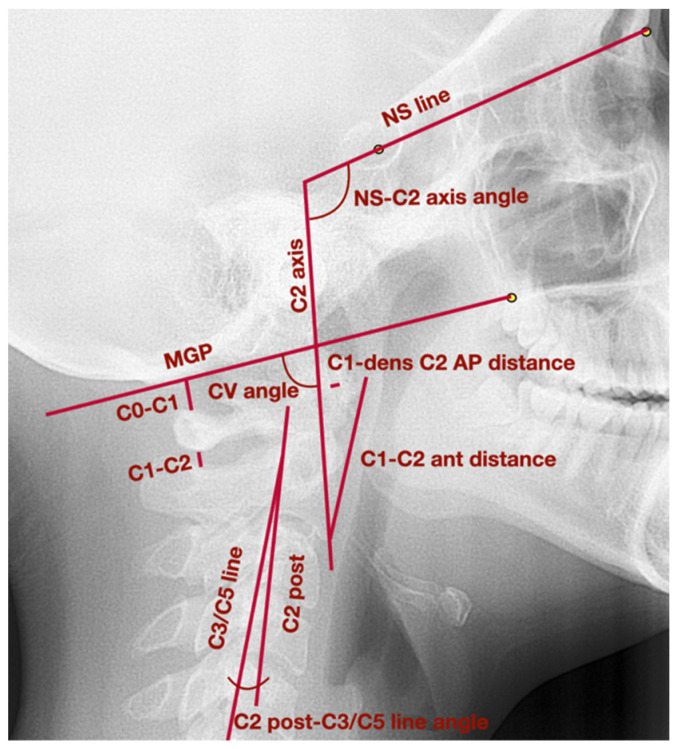
Lateral cephalogram with marked points, lines, and angles presented in Table 1 used to assess head position, cervical vertebrae, and functional spaces. AP—anteroposterior; CV angle—craniovertebral angle; MGP—McGregor’s Plane; NS—nasion-sella line; C0—basiocciput; C1—C3; C5—first, second, third, fifth cervical vertebrae.

Figure 2 presents lateral cephalogram with marked points, lines, and angles presented in Table 1 used to assess head position, cervical vertebrae, and functional spaces.

### 2.5. Sample Size Calculation

The sample size was estimated on the basis of an initial pilot study. We considered a power of 80% and a probability of the type I error 0.05. At least 15 patients were necessary in order to detect a 20% difference in Wits measurement after the end of the treatment.

### 2.6. Statistical Analysis

Statistica 13.0 software (Dell Inc., Aliso Viejo, CA, USA) was used to perform all data analyses. There were calculated: mean differences, standard deviations, 95% confidence interval (95% CI), and mean percentage changes between the values obtained before and after the end of the treatment. To check whether the differences before and after the end of the treatment were statistically significant the following tests were applied: T-Student test and Wilcoxon test. The statistical significance level was set at *p* = 0.05.

## 3. Results

### 3.1. Flow of Participants

A total of 44 patients diagnosed with TMD met the inclusion criteria and were included in the study. However, four patients resigned from the treatment during the first month of the occlusal splint therapy, because they were not able to wear the occlusal splint day and night. There were 32 women (80.0%) and 8 men (20.0%) who participated in the study. The average age was 28.4 ± 10.2 years old.

There were 15 patients included in the control group: 12 women (80.0%) and 3 men (20.0%). The average age was: 31.3 ± 12.9 years old. There were no statistically significant differences between the study group and the control group regarding the sex (*p* = 1.000) and the age (*p* = 0.217) of the participants.

**Figure 3:**
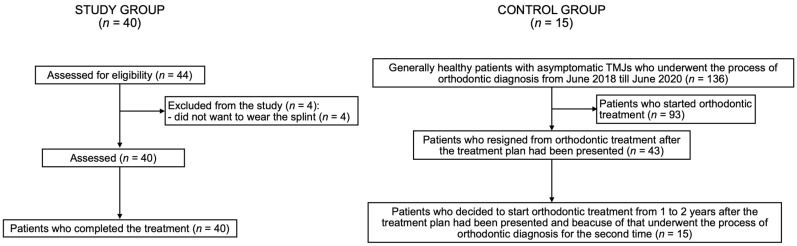
Flow of the participants during the study. TMJ—temporomandibular joint; TMD—temporomandibular joint disorders.

Figure 3 presents flow of the participants during the study.

The vast majority of the examined patients from the study group were diagnosed with myalgia (29 patients, 72.5%), whereas arthralgia was diagnosed in 11 patients (32.5%).

Myalgia or arthralgia had lasted from 2.5 to 6 months in the examined patients before they came for the orthodontic consultation. Headache attributed to TMD had lasted from 6 to 24 months before the initial orthodontic examination. However, none of the patients realized that the headache they suffered from was in fact attributed to TMD. It was impossible to assess the duration of disc displacement with reduction. Only 7 out of 28 patients with disc displacement with reduction knew they had reciprocal clicking in their TMJs before the initial examination, whereas the remaining 21 patients heard the reciprocal clicking within their TMJs for the first time during the physical examination. Those patients who were aware of the joint sounds within TMJs, reported that the reciprocal clicking lasted for a longer period of time (more than 1 year), but they were not able to assess the exact period of time. It was impossible to assess the duration time of degenerative joint disease and joint subluxation, because none of the diagnosed patients had ever been informed by other practitioners about those diagnoses.

Table 2 presents the frequency of different diagnoses on the basis of DC/TMD among the examined patients within the study group.

### 3.2. Research Question

After the end of the treatment, patients diagnosed with TMD reported significant TMJ pain reduction (*p* < 0.0001), and the average value of maximum mouth opening was significantly increased (*p* = 0.0011). The average values of maximum mouth opening and pain scores before and after the end of the treatment were presented in a previously published manuscript [20].

Having compared the examined parameters within the cervicofacial skeleton during the initial examination between the study group and the control group, it was found that only the skeletal vertical dimension was significantly higher in the study group (25.5 ± 7.4°) compared to the control group (21.1 ± 5.5°) (*p* = 0.0469).

Table 3 presents the comparison of the examined parameters within the cervicofacial skeleton during the initial examination between the study group and the control group.

Within the control group, there were no statistically significant differences between the first and the second examination regarding: the vertical and sagittal position of the mandible, head and cervical vertebrae positions, and the average size of cervical vertebrae functional spaces.

Table 4 presents the assessment of changes in the cervicofacial skeleton that occurred between the first and the second orthodontic examination within the control group.

In case of patients diagnosed with TMD who underwent occlusal splint therapy combined with physiotherapy, the average value of vertical dimension, measured as the angle between the bases of maxilla and the mandible (NL-ML angle), significantly (*p* < 0.0001) increased from 25.5 ± 7.4° (95% CI 23.2 to 27.6) to 27.1 ± 7.8° (95% CI 24.2 to 29.8) after the end of the therapy. The sagittal position of the mandible became more retruded after the treatment. The average value of Wits measurement increased by 46.2% from 1.3 ± 2.9 mm (95% CI 0.2 to 2.2) to 1.9 ± 3.2 mm (95% CI 0.8 to 3.0) (*p* = 0.0065), and at the same time the average value of the ANB angle increased by 15.79% from 3.8 ± 2.8° (95% CI 2.9 to 4.7) to 4.4 ± 3.2° (95% CI 3.3 to 5.5) (*p* = 0.0092) at the end of the treatment.

Moreover, the angle C2 post-C3/C5 line angle increased by 43.1% from 10.6 ± 7.9 mm (95% CI 8.5 to 13.5) to 14.6 ± 9.1 mm (95% CI 11.5 to 17.6) (*p* < 0.0001) at the end of the treatment. The craniovertebral angle, the angle between the cranial base and the long axis of C2, the distance between the odontoid process and C1, as well as the distance between the anterior surfaces of C1 and C2 did not change significantly after the end of the treatment. Only the functional space between C1 and C2 significantly decreased by 10.17% from 5.9 ± 2.0 mm (95% CI 5.4 to 6.3) to 5.3 ± 1.8 mm (95% 4.7 to 6.1) (*p* = 0.0042). The height of the functional space between C0 and C1 remained unchanged after the treatment.

Table 5 presents the craniovertebral and craniomandibular changes in cephalometric images after occlusal splint therapy combined with physiotherapy in patients diagnosed with TMD.

## 4. Discussion

Occlusal splint therapy and physiotherapy are considered to be minimally invasive, effective, and first-choice methods of treatment of TMD-related pain [24]. Both of the methods have been found to increase mandibular range of motion [13]. Ismail et al. [25] noticed that in the 3-month observations none of these methods appeared to be preferable. Pficer et al. [14] stated that occlusal splints play a significant role in the treatment of TMD in the short term, whereas in the long-term observations they are not superior to any other methods of treatment. Manipulation or mobilization of the upper cervical area appeared to be very effective in reducing pain related to TMD and in increasing maximum mouth opening [15]. The most recent review and meta-analysis by Zhang et al. [26], which compared the effects of occlusal splint therapy and physiotherapy, stated that there is no high-quality evidence to distinguish between the effectiveness of the physiotherapy and that of occlusal splint therapy.

Although there are many studies in which patients with TMD were treated with either physiotherapy or occlusal splints, the number of studies in which physiotherapy and occlusal splint therapy are combined is very limited. Lopez et al. [27] found that a treatment protocol based on manual therapy with occlusal splint therapy was more effective in patients diagnosed with TMD than occlusal splint therapy performed alone.

According to our study, physiotherapy and occlusal splint therapy combined together led to some statistically significant changes within the cervicofacial skeleton. Because of the fact that additional lateral X-rays were not taken after the initial series of physiotherapy and before the onset of occlusal splint therapy combined with physiotherapy, it is impossible to distinguish what is the exact cause of the observed changes: only physiotherapy, only occlusal splint therapy, or the combination of both of the abovementioned methods of treatment.

After the end of the combined physiotherapy with the occlusal splint therapy, the skeletal vertical dimension was significantly increased. It was caused by two mechanisms: primarily by posterior rotation of the mandible, and secondly by posterior rotation of the cranium. The latter played a minor role. Posterior cranium rotation was probably limited thanks to the manual therapy which aimed at anterior cranium rotation and cervical lordosis restoration throughout the entire period of treatment time. No negative compensation mechanisms were observed in cervical spine and head posture due to the increased skeletal vertical dimension. The cervical lordosis was restored in many patients after the end of the treatment. It may be speculated that significant increase of skeletal vertical dimension was caused by the occlusal splint therapy, whereas physiotherapy led to the cervical lordosis restoration and limited posterior cranium rotation.

Unfortunately, the sagittal position of the mandible became more retruded in the majority of cases. Posterior rotation of mandible moves point B downward and backward, and therefore the mandible becomes more retruded. Our former study [20] also confirmed posterior rotation of the mandible. We have found that the mandibular condyles did not significantly change their sagittal positions within the glenoid fossae, but they were mostly clockwise-rotated after the end of the treatment combining occlusal splint therapy and physiotherapy [20]. Therefore, there may arise a question, what causes the mandible to be positioned posteriorly after the long-term (6 months) occlusal splint therapy. On the one hand, posterior rotation of the mandible may be indeed the so-called orthopedically stable position in which further occlusal treatments (orthodontics and/or prosthodontics) should be performed. However, on the other hand, it must be considered that the posterior rotation of the mandible may be the side effect of the long-term occlusal splint therapy. It should be noted that during long-term occlusal splint therapy with the occlusal splints worn day and night, some negative changes (i.e., muscle contracture) may also develop within the muscles, and therefore the mandible may have a limited range of anterior rotation. It must be remembered that the occlusal splints, even the thinnest ones, cause difficulties with lip closure. This problem is mostly noticeable in patients with anterior open bite; however, it occurs in all patients. To close the lips with an occlusal splint inside the mouth, patients must increase the tension in the lips and therefore they activate two very strong muscles, namely: the superior and middle pharyngeal constrictor muscles. Both of these muscles move the mandible back either directly (superior pharyngeal constrictor muscle) or indirectly (middle pharyngeal constrictor muscle, which is attached to the hyoid bone).

Hyperactivity of the superior pharyngeal constrictor muscle may significantly affect the position of all of the anatomical structures it is attached to, namely: cranium, mandible, pterygomandibular raphe, tongue, and cervical spine [19]. Four different parts of the superior pharyngeal constrictor muscle can be distinguished depending on the point of origin.

The most superior part, known as the pterygopharyngeal part, is attached to the posterior margin of the medial pterygoid plate of the sphenoid bone, as well as to the pterygoid hamulus of the sphenoid bone [19]. Hyperactivation of this part of the muscle moves the pterygoid processes back and therefore the whole cranium rotates counterclockwise, and the craniovertebral angle decreases. In our study, we observed that the average values of the craniovertebral angle were insignificantly lower after the end of the treatment. However, it must be noted that all of the patients underwent the physiotherapy which focused, among other things, on the anterior rotation of the cranium. It may be speculated that if the patients had been treated only with the occlusal splints, without physiotherapy, the average value of the craniovertebral angle after the end of the treatment would have been significantly lower. Moreover, whenever the cranium rotates, the inclination of the maxilla changes. With the increased activation of the superior pharyngeal constrictor muscle, the posterior part of the maxilla goes down, whereas the anterior part goes up. This means the maxilla also rotates counterclockwise and as a consequence the bite opens.

The second part of the superior pharyngeal constrictor muscle, known as the buccopharyngeal part, is attached to the pterygomandibular raphe. The pterygomandibular raphe connects the superior pharyngeal constrictor muscle with the buccinator muscle [19]. Hyperactivation of the buccopharyngeal part of the superior pharyngeal constrictor muscle increases the tension in the buccinator muscle through the pterygomandibular raphe. Some of the buccinator muscle fibers become the origin of the orbicularis oris muscle. Consequently, increased tension in the superior pharyngeal constrictor muscle may increase the lip pressure on the anterior border of the mandible. This may promote posterior rotation (clockwise rotation) of the mandible. Moreover, increased tension in buccinator muscles may hypothetically increase the pressure on the dental arches from the outside to the inside of the oral cavity. The transverse dimension of the dental arch with the occlusal splint remains unchanged because the acrylic occlusal splint protects the shape of the dental arch against the tension in buccinator muscles. However, the opposite dental arch may present the decreased transverse dimension after the end of the long-term occlusal splint therapy due to the lingual crown tipping of posterior teeth within the “non-protected” dental arch. This may have another significant implication, namely the changes in the positions of buccal cusps of posterior teeth may increase the vertical dimension, and therefore may also cause the anterior open bite. This issue will be discussed in detail in our further studies.

The mylopharyngeal part of the superior pharyngeal constrictor muscle is attached directly to the mandible, most commonly on the posterior part of the mylohyoid line, and less frequently away from the mylohyoid line [19]. In case of the increased activity of the mylopharyngeal part of the superior pharyngeal constrictor muscle, the mandible is pulled back and down, and therefore it rotates clockwise. Clockwise rotation of the mandible causes anterior open bite due to the increased vertical dimension.

Finally, the lowest part of the superior pharyngeal constrictor muscle, known as the glossopharyngeal part, is attached to the root of the tongue [19]. This muscle goes along with the styloglossus and palatoglossus. Its fibers also either merge or interlace with the muscle fibers of the mylopharyngeal part [19]. Increased tension within the superior pharyngeal constrictor muscle may limit the anterior movement of the tongue, may hypothetically lead to the low tongue posture, and finally may contribute to tongue thrusting.

The superior pharyngeal constrictor muscle has four (described above) points of the origin, and only one point of the insertion, which is the posterior wall of the pharynx. In the upper part, the superior pharyngeal constrictor muscle is attached to the pharyngeal tubercle on the occipital bone. This is the second direct connection between this muscle and the cranium. Changes within the tension of the superior pharyngeal constrictor muscle may therefore affect the craniovertebral angle, as described earlier.

From these very possible and logical sequences of cause and effect arises the fundamental question of what really happens within the structure of the superior and middle pharyngeal constrictor muscles throughout the long-term occlusal splint therapy and how these changes affect the position and motion of the mandible, cranium, tongue, and cervical spine?

It has been found that the hyperextension of the mandible may cause the superior pharyngeal constrictor muscle syndrome. Several symptoms of superior pharyngeal constrictor muscle syndrome have been listed, including: throat pain, pain in the area of TMJ, pain in the temporal area, pain during swallowing, otalgia, and finally problems with coordination of swallowing reflex [28]. Some of the patients who are treated with the occlusal splints for a few months and have not suffered from any symptoms of TMD for a longer period of time suddenly start reporting some of the abovementioned symptoms. When this happens, it must be thoroughly evaluated whether these are the recurring symptoms of TMD or they are related to negative changes within the superior pharyngeal constrictor muscle.

Having taken all of the abovementioned into consideration, it seems necessary for the physiotherapists to stretch the pterygomandibular raphe during the entire time of occlusal splint therapy to decrease the tension of the superior pharyngeal constrictor muscle.

The above-discussed craniovertebral and craniomandibular changes did not occur in individuals from the control group. This clearly shows that the observed changes within the cervicofacial skeleton were the effect of the treatment combining physiotherapy with occlusal splint therapy, and that they do not appear over time in healthy individuals.

Moya et al. [29] noticed that increasing occlusal vertical dimension with occlusal splint worn only for one hour led to significant extension of the head on the cervical spine, as well as to decrease of the cervical spine lordosis. Miralles et al. [30] found that increasing occlusal vertical dimension in children led to cervical dysfunction. Oliveira et al. [31] confirmed that occlusal splint therapy combined with physiotherapeutic exercises significantly improved postural balance.

Orthopedic stability of TMJs is highly dependent on the stability of craniovertebral joints. The unstable position of craniovertebral joints affects the three-dimensional position of TMJs’ articular fossae. Condyles posteriorly displaced in articular fossae should not be treated primarily by mandibular advancement, but by evaluation and correction of the mobility of craniovertebral joints, and by anterior rotation of the cranium. Anterior rotation of the head changes the position of the glenoid fossa of the temporal bone, leads to opening posterior joint space, and at same time stabilizes the articular fossae on the articular discs. Therefore, treatment of patients diagnosed with TMD should primarily aim at achieving orthopedic stability in craniovertebral joints, and secondly in synovial TMJs.

TMD is often related to pain in the area of the head and neck. Among different types of headaches, cervicogenic headache should be of great interest for physiotherapists and dentists. It is caused by disorders in the area of the cervical spine, including: bony structures and disc and soft tissue components [32]. The upper cervical nociceptive afferents, especially from the area of C1, C2, and C3, converge with the trigeminal nociceptive afferents. As a consequence, pain originating from the upper cervical nerves may be transferred to the area innervated by the trigeminal nerve [33,34]. Therefore, the cervical functional spaces, especially between basiocciput and C1, as well as C1 and C2, must be examined thoroughly by the physiotherapist. The normal functional spaces should measure 6.5 ± 2.5 mm [18]. In the case of decreased spaces, their width should be restored.

Cervical lordosis is a natural curvature of the cervical spine. It is important not only for supporting physiological loads, but also to keep the cervical spine stable with joints functioning without pain and friction [35]. Cervical lordosis is also necessary to support physiological functions, including mastication, breathing, making sounds, and eye movement. There has also been found an association between TMD and a loss of cervical lordosis [36]. It is important to mobilize cervical vertebrae to achieve a physiological range of motion before the lordosis is restored.

The presented study has several strengths. First of all, this is the first study which prospectively analyzed the craniovertebral and craniomandibular changes which occurred in patients with TMD after the end of the treatment combining physiotherapy and occlusal splint therapy. Secondly, the methodology is comprehensive, and the results answer the question of what happens within the facial and cervical skeleton after the end of the treatment. Finally, this study, as the first one, indicates the possible relationship between increased skeletal vertical dimension and the hyperactivity of the superior pharyngeal constrictor muscle. 

On the other hand, there are also a few weaknesses of this study. Firstly, it is impossible to state which effects observed within the cervicofacial skeleton were caused only by physiotherapy, only by occlusal splint therapy, and finally were caused by the combination of physiotherapy and occlusal splint therapy. We did not take additional lateral X-rays after the end of initial series of physiotherapy, because the study was performed with respect to ALARA principle and we did not want to increase radiation to patients. Secondly, we did not measure the distance between the two anthropometric points on patients’ faces to assess the changes within the vertical dimension. Cephalometric assessment of the lower floor of the face is approximate. Thirdly, we did not perform among the examined patients any tests to diagnose the autoimmune etiology of TMD. It may be speculated that the effects of TMD treatment in patients with and without autoimmune diseases may differ and should be thoroughly evaluated in further studies. Fourthly, the control group was limited. This was because we included into the control group only the generally healthy patients with asymptomatic TMJs who had undergone orthodontic diagnosis twice, which was explained earlier in the text. Finally, we did not describe all the changes that occur within the muscles of head and neck after the manual therapy, because it seems to us impossible to be explained due to the large number of connections and interactions between different muscles. To sum up, the major limitations of the study were: the lack of additional radiographic diagnosis after the end of the initial series of physiotherapy, the limited study sample, and lack of diagnosis of autoimmune diseases.

## 5. Conclusions

Occlusal splint therapy and physiotherapy combined together significantly affect: the vertical and sagittal position of mandible, as well as the width of functional space between C1 and C2. Cooperation of physiotherapists with dental practitioners is necessary in the treatment of patients with TMD, including TMJ osteoarthritis. Further studies should answer the question what changes occur within the superior and middle pharyngeal constrictor muscles after long-term occlusal splint therapy with occlusal splints worn day and night.

## Figures and Tables

**Table 1:** Reference points, lines, and angles used in cephalometric analysis to assess the changes in cervicofacial skeleton after occlusal splint therapy combined with physiotherapy in patients diagnosed with temporomandibular joint disorders [22,23].

Measurement	Point/Line/Angle	Description
General points and lines	Point A	Subspinale—point localized in the deepest area of the anterior outline of the maxilla, below the anterior nasal spine
	Point B	Supramentale—point localized in the deepest area of the anterior outline of the mandible, above the pogonion
	Point S	Sella—geometrical center of Sella turcica
	Point Pg	Pogonion—the most prominent point localized in the mental tuberosity
	Point N	Nasion—the most anterior point localized in the frontonasal suture
	C2 axis	Long axis of C2 vertebra, which crosses the apex of the odontoid process and the most inferior anterior angle of the body of the second cervical vertebra
	C2 post	Line tangent to the posterior wall of the odontoid process of the second cervical vertebra
	C3/C5 line	Line which links the most posterior superior angle of the body of the third cervical vertebra with the most posterior inferior angle of the body of the fifth cervical vertebra
	MGP	McGregor’s Plane—line which links posterior nasal spine with the basiocciput
	NS line	Line which crosses the points: nasion and sella
	NL line	Nasal line—line which crosses the points: anterior nasal spine and posterior nasal spine
	ML line	Mandibular line—line tangent to the lower border of the mandible, which crosses points: gnathion and the lowest point in the masseteric tuberosity
	NA line	Line which crosses points: nasion and point A
	NB line	Line which crosses points: nasion and point B
Vertical position of mandible	NL-ML angle	The angle between NL line and ML line
Sagittal position of mandible	Wits	The distance between the perpendicular projection of points A and B onto the functional occlusal plane
	ANB	The angle between the lines: NA line and NB line
Head position and cervical vertebrae	CV angle	The posterior angle between the lines: C2 axis and MGP
	C2 post-C3/C5 line angle	The angle between the lines: posterior wall of C2 odontoid process and C3/C5 line; the value of the angle is positive when the C2 axis is placed anteriorly to C3/C5 line; when the C2 axis is placed posteriorly to C3/C5 line, the value of the angle is negative
	C1-dens C2 AP distance	The anteroposterior distance between anterior surface of the odontoid process and posterior border of the atlas anterior arch
	C1-C2 ant distance	The distance between the most anterior point of the atlas anterior arch and the most inferior anterior angle of the body of the second cervical vertebra
	NS-C2 axis angle	The anterior angle between the lines: NS line and C2 axis
Cervical vertebrae functional spaces	C0-C1 distance	The distance between basiocciput and superior part of the posterior arch of the atlas
	C1-C2 distance	The distance between inferior part of the posterior arch of the atlas and superior part of the spinous process of the second cervical vertebra

AP—anteroposterior; CV—craniovertebral; LO—lower; UP—upper.

**Table 2:** The frequency of different diagnoses on the basis of DC/TMD among the examined patients within the study group.

Diagnosis on the Basis of DC/TMD	Number of Patients (%)
Myalgia	29 (72.5%)
Arthralgia	11 (27.5%)
Headache attributed to TMD	13 (32.5%)
Disc displacement with reduction	31 (77.5%)
Disc displacement with reduction with intermittent locking	3 (7.5%)
Disc displacement without reduction with limited opening	0
Disc displacement without reduction without limited opening	0
Degenerative joint disease	5 (12.5%)
Subluxation	11 (27.5%)

**Table 3:** The comparison of the examined parameters within the cervicofacial skeleton during the initial examination between the study group and the control group.

Comparable Characteristic		Study Group av. ± SD (95% CI)	Control Group av. ± SD (95% CI)	*p*-Value
Vertical position of mandible	NL-ML angle (°)	25.5 ± 7.4 (23.2 to 27.6)	21.1 ± 5.5 (18.0 to 24.1)	0.0469 ^a^
Sagittal position of mandible	Wits (mm)	1.3 ± 2.9 (0.2 to 2.2)	0.6 ± 2.8 (−0.9 to 2.2)	0.5278 ^a^
	ANB (°)	3.8 ± 2.8 (2.9 to 4.7)	2.3 ± 2.7 (0.9 to 3.8)	0.0932 ^a^
Head position and cervical vertebrae	CV angle (°)	100.3 ± 8.3 (97.6 to 103.0)	100.4 ± 7.1 (96.4 to 104.3)	0.9478 ^a^
	C2 post-C3/C5 line angle (°)	10.6 ± 7.9 (8.5 to 13.5)	9.0 ± 7.7 (4.7 to 13.2)	0.4845 ^a^
	C1-dens C2 AP distance (mm)	1.5 ± 0.3 (1.4 to 1.6)	1.5 ± 0.2 (1.4 to 1.6)	0.6638 ^b^
	C1-C2 ant distance (mm)	33.3 ± 3.5 (32.2 to 34.5)	34.4 ± 2.6 (32.9 to 35.9)	0.2964 ^a^
	NS-C2 axis angle (°)	87.6 ± 8.3 (85.2 to 90.7)	87.3 ± 6.5 (83.7 to 90.9)	0.8130 ^a^
Cervical vertebrae functional spaces	C0-C1 distance (mm)	6.8 ± 3.2 (5.8 to 7.6)	7.4 ± 1.6 (6.5 to 8.2)	0.3443 ^a^
	C1-C2 distance (mm)	5.9 ± 2.0 (5.4 to 6.3)	5.5 ± 1.7 (4.6 to 6.5)	0.4368 ^a^

^a^ *t*-Student test; ^b^ U Mann–Whitney test.

**Table 4:** Assessment of changes in the cervicofacial skeleton that occurred between the first and the second orthodontic examination in the control group.

Comparable Characteristic		First Examination av. ± SD (95% CI)	Second Examination (1–2 Years after the Initial One) av. ± SD (95% CI)	*p*-Value
Vertical position of mandible	NL-ML angle (°)	21.1 ± 5.5 (18.0 to 24.1)	21.1 ± 5.5 (18.0 to 24.1)	1.0000 ^a^
Sagittal position of mandible	Wits (mm)	0.6 ± 2.8 (−0.9 to 2.2)	0.6 ± 2.8 (−0.9 to 2.2)	1.0000 ^a^
	ANB (°)	2.3 ± 2.7 (0.9 to 3.8)	2.3 ± 2.7 (0.9 to 3.8)	1.0000 ^b^
Head position and cervical vertebrae	CV angle (°)	100.4 ± 7.1 (96.4 to 104.3)	100.4 ± 7.0 (96.5 to 104.2)	0.3003 ^b^
	C2 post-C3/C5 line angle (°)	9.0 ± 7.7 (4.7 to 13.2)	9.0 ± 7.6 (4.7 to 13.2)	0.5563 ^b^
	C1-dens C2 AP distance (mm)	1.5 ± 0.2 (1.4 to 1.6)	1.5 ± 0.2 (1.4 to 1.6)	0.5930 ^b^
	C1-C2 ant distance (mm)	34.4 ± 2.6 (32.9 to 35.9)	34.4 ± 2.6 (33.0 to 35.8)	0.3505 ^b^
	NS-C2 axis angle (°)	87.3 ± 6.5 (83.7 to 90.9)	87.3 ± 6.5 (83.7 to 90.9)	0.9029 ^a^
Cervical vertebrae functional spaces	C0-C1 distance (mm)	7.4 ± 1.6 (6.5 to 8.2)	7.4 ± 1.6 (6.5 to 8.2)	0.8494 ^a^
	C1-C2 distance (mm)	5.5 ± 1.7 (4.6 to 6.5)	5.5 ± 1.6 (4.6 to 6.4)	0.8939 ^b^

^a^ *t*-Student test; ^b^ Wilcoxon test.

**Table 5:** Assessment of the craniovertebral and craniomandibular changes in cephalometric images after occlusal splint therapy combined with physiotherapy in patients diagnosed with temporomandibular joint disorders.

Comparable Characteristic		Before Treatment av. ± SD (95% CI)	After Treatment av. ± SD (95% CI)	*p*-Value
Vertical position of mandible	NL-ML angle (°)	25.5 ± 7.4 (23.2 to 27.6)	27.1 ± 7.8 (24.2 to 29.8)	<0.0001 ^a^
Sagittal position of mandible	Wits (mm)	1.3 ± 2.9 (0.2 to 2.2)	1.9 ± 3.2 (0.8 to 3.0)	0.0065 ^a^
	ANB (°)	3.8 ± 2.8 (2.9 to 4.7)	4.4 ± 3.2 (3.3 to 5.5)	0.0092 ^b^
Head position and cervical vertebrae	CV angle (°)	100.3 ± 8.3 (97.6 to 103.0)	99.7 ± 9.4 (96.6 to 103.1)	0.4735 ^a^
	**C2 post-C3/C5 line angle (°)**	**10.6 ± 7.9 (8.5 to 13.5)**	**14.6 ± 9.1 (11.5 to 17.6)**	**<0.0001 ^a^**
	C1-dens C2 AP distance (mm)	1.5 ± 0.3 (1.4 to 1.6)	1.5 ± 0.3 (1.4 to 1.6)	0.5255 ^b^
	C1-C2 ant distance (mm)	33.3 ± 3.5 (32.2 to 34.5)	33.3 ± 3.32 (32.2 to 34.1)	0.9406 ^b^
	NS-C2 axis angle (°)	87.6 ± 8.3 (85.2 to 90.7)	88.0 ± 8.6 (85.2 to 90.8)	0.8265 ^a^
Cervical vertebrae functional spaces	C0-C1 distance (mm)	6.8 ± 3.2 (5.8 to 7.6)	6.8 ± 3.1 (5.8 to 7.9)	0.3245 ^b^
	**C1-C2 distance (mm)**	**5.9 ± 2.0 (5.4 to 6.3)**	**5.3 ± 1.8 (4.7 to 6.1)**	**0.0042 ^a^**

^a^ *t*-Student test; ^b^ Wilcoxon test.

## Data Availability

The data underlying this article are available in the article.

## References

[1] Schiffman E., Ohrbach R., Truelove E., Look J., Anderson G., Goulet J.P., List T., Svensson P., Gonzalez Y., Lobbezoo F. (2014). International RDC/TMD Consortium Network, International association for Dental Research; Orofacial Pain Special Interest Group, International Association for the Study of Pain. Diagnostic Criteria for Temporomandibular Disorders (DC/TMD) for Clinical and Research Applications: Recommendations of the International RDC/TMD Consortium Network and Orofacial Pain Special Interest Group. J. Oral Facial. Pain Headache.

[2] Michelotti A., Alstergren P., Goulet J.P., Lobbezoo F., Ohrbach R., Peck C., Schiffman E., List T. (2016). Next steps in development of the diagnostic criteria for temporomandibular disorders (DC/TMD): Recommendations from the International RDC/TMD Consortium Network workshop. J. Oral Rehabil..

[3] Gauer R.L., Semidey M.J. (2015). Diagnosis and treatment of temporomandibular disorders. Am. Fam. Physician.

[4] Liu F., Steinkeler A. (2013). Epidemiology, diagnosis, and treatment of temporomandibular disorders. Dent. Clin. N. Am..

[5] Valesan L.F., Da-Cas C.D., Réus J.C., Denardin A.C.S., Garanhani R.R., Bonotto D., Januzzi E., de Souza B.D.M. (2021). Prevalence of temporomandibular joint disorders: A systematic review and meta-analysis. Clin. Oral Investig..

[6] Robinson J.L., Johnson P.M., Kister K., Yin M.T., Chen J., Wadhwa S. (2020). Estrogen signaling impacts temporomandibular joint and periodontal disease pathology. Odontology.

[7] Manfredini D., Guarda-Nardini L., Winocur E., Piccotti F., Ahlberg J., Lobbezoo F. (2011). Research diagnostic criteria for temporomandibular disorders: A systematic review of axis I epidemiologic findings. Oral Surg. Oral Med. Oral Pathol. Oral Radiol. Endodontol..

[8] Manfredini D., Lombardo L., Siciliani G. (2017). Temporomandibular disorders and dental occlusion. A systematic review of association studies: End of an era?. J. Oral Rehabil..

[9] Magnusson T., Egermarki I., Carlsson G.E. (2005). A prospective investigation over two decades on signs and symptoms of temporomandibular disorders and associated variables. A final summary. Acta Odontol. Scand..

[10] Michelotti A., Iodice G. (2010). The role of orthodontics in temporomandibular disorders. J. Oral Rehabil..

[11] Derwich M., Mitus-Kenig M., Pawlowska E. (2020). Interdisciplinary Approach to the Temporomandibular Joint Osteoarthritis-Review of the Literature. Medicina.

[12] Bialosky J.E., Bishop M.D., Price D.D., Robinson M.E., George S.Z. (2009). The mechanisms of manual therapy in the treatment of musculoskeletal pain: A comprehensive model. Man. Ther..

[13] Gomes C.A., Politti F., Andrade D.V., de Sousa D.F., Herpich C.M., Dibai-Filho A.V., Gonzalez Tde O., Biasotto-Gonzalez D.A. (2014). Effects of massage therapy and occlusal splint therapy on mandibular range of motion in individuals with temporomandibular disorder: A randomized clinical trial. J. Manip. Physiol. Ther..

[14] Kuzmanovic Pficer J., Dodic S., Lazic V., Trajkovic G., Milic N., Milicic B. (2017). Occlusal stabilization splint for patients with temporomandibular disorders: Meta-analysis of short and long term effects. PLoS ONE.

[15] Calixtre L.B., Moreira R.F., Franchini G.H., Alburquerque-Sendín F., Oliveira A.B. (2015). Manual therapy for the management of pain and limited range of motion in subjects with signs and symptoms of temporomandibular disorder: A systematic review of randomised controlled trials. J. Oral Rehabil..

[16] Lopez A.J., Scheer J.K., Leibl K.E., Smith Z.A., Dlouhy B.J., Dahdaleh N.S. (2015). Anatomy and biomechanics of the craniovertebral junction. Neurosurg. Focus.

[17] Akita K., Sakaguchi-Kuma T., Fukino K., Ono T. (2019). Masticatory Muscles and Branches of Mandibular Nerve: Positional Relationships Between Various Muscle Bundles and Their Innervating Branches. Anat. Rec..

[18] Shaw S.M., Martino R., Mahdi A., Sawyer F.K., Mathur S., Hope A., Agur A.M. (2017). Architecture of the Suprahyoid Muscles: A Volumetric Musculoaponeurotic Analysis. J. Speech Lang. Hear. Res..

[19] Tsumori N., Abe S., Agematsu H., Hashimoto M., Ide Y. (2007). Morphologic characteristics of the superior pharyngeal constrictor muscle in relation to the function during swallowing. Dysphagia.

[20] Derwich M., Pawlowska E. (2022). Do the Mandibular Condyles Change Their Positions within Glenoid Fossae after Occlusal Splint Therapy Combined with Physiotherapy in Patients Diagnosed with Temporomandibular Joint Disorders? A Prospective Case Control Study. J. Pers. Med..

[21] Rocabado M., Iglarsh Z.A. (1991). Physical modalities and manual techniques used in the treatment of maxillofacial pain. Musculoskeletal Approach to Maxillofacial Pain.

[22] Derwich M., Mitus-Kenig M., Pawlowska E. (2021). Is the Temporomandibular Joints' Reciprocal Clicking Related to the Morphology and Position of the Mandible, as Well as to the Sagittal Position of Lower Incisors?-A Case-Control Study. Int. J. Environ. Res. Public Health.

[23] Rocabado M. (1983). Biomechanical relationship of the cranial, cervical, and hyoid regions. J. Craniomandib. Pract..

[24] Wieckiewicz M., Boening K., Wiland P., Shiau Y.Y., Paradowska-Stolarz A. (2015). Reported concepts for the treatment modalities and pain management of temporomandibular disorders. J. Headache Pain..

[25] Ismail F., Demling A., Hessling K., Fink M., Stiesch-Scholz M. (2007). Short-term efficacy of physical therapy compared to splint therapy in treatment of arthrogenous TMD. J. Oral Rehabil..

[26] Zhang L., Xu L., Wu D., Yu C., Fan S., Cai B. (2021). Effectiveness of exercise therapy versus occlusal splint therapy for the treatment of painful temporomandibular disorders: A systematic review and meta-analysis. Ann. Palliat. Med..

[27] Espí-López G.V., Arnal-Gómez A., Cuerda Del Pino A., Benavent-Corai J., Serra-Añó P., Inglés M. (2020). Effect of Manual Therapy and Splint Therapy in People with Temporomandibular Disorders: A Preliminary Study. J. Clin. Med..

[28] Shankland W.E. (2010). Anterior throat pain syndromes: Causes for undiagnosed craniofacial pain. Cranio.

[29] Moya H., Miralles R., Zuñiga C., Carvajal R., Rocabado M., Santander H. (1994). Influence of stabilization occlusal splint on craniocervical relationships. Part I: Cephalometric analysis. Cranio.

[30] Miralles R., Moya H., Ravera M.J., Santander H., Zúñiga C., Carvajal R., Yazigi C. (1997). Increase of the vertical occlusal dimension by means of a removable orthodontic appliance and its effect on craniocervical relationships and position of the cervical spine in children. Cranio.

[31] Oliveira S.S.I., Pannuti C.M., Paranhos K.S., Tanganeli J.P.C., Laganá D.C., Sesma N., Duarte M., Frigerio M.L.M.A., Cho S.C. (2019). Effect of occlusal splint and therapeutic exercises on postural balance of patients with signs and symptoms of temporomandibular disorder. Clin. Exp. Dent. Res..

[32] Headache Classification Committee of the International Headache Society (IHS) (2013). The International Classification of Headache Disorders, 3rd edition (beta version). Cephalalgia.

[33] Barmherzig R., Kingston W. (2019). Occipital Neuralgia and Cervicogenic Headache: Diagnosis and Management. Curr. Neurol. Neurosci. Rep..

[34] Bogduk N. (2001). Cervicogenic headache: Anatomic basis and pathophysiologic mechanisms. Curr. Pain. Headache Rep..

[35] Rocabado M. (1987). The importance of soft tissue mechanics in stability and instability of the cervical spine: A functional diagnosis for treatment planning. Cranio.

[36] Been E., Shefi S., Soudack M. (2017). Cervical lordosis: The effect of age and gender. Spine J..

